# Fibrous Dysplasia versus Juvenile Ossifying Fibroma: A Dilemma

**DOI:** 10.1155/2016/6439026

**Published:** 2016-12-22

**Authors:** Sreelakshmi N. Nair, Raghavendra Kini, Prasanna Kumar Rao, Gowri P. Bhandarkar, Roopashri Rajesh Kashyp, Manjunath Rai, Neel Naik, Athul Santhosh

**Affiliations:** ^1^Department of Oral Medicine and Radiology, AJ Institute of Dental Sciences, Mangalore, Karnataka, India; ^2^Department of Oral and Maxillofacial Surgery, AJ Institute of Dental Sciences, Mangalore, Karnataka, India

## Abstract

Fibrous dysplasia (FD) is a condition characterized by excessive proliferation of bone forming mesenchymal cells which can affect one bone (monostotic type) or multiple bones (polyostotic type). It is predominantly noticed in adolescents and young adults. Fibrous dysplasia affecting the jaws is an uncommon condition. The most commonly affected facial bone is the maxilla, with facial asymmetry being the chief complaint. The lesion in many instances is confused with ossifying fibroma (OF). Diagnosis of these two lesions has to be done based on clinical, radiographic, and microscopic findings. Here, we present a case of fibrous dysplasia of maxilla in a nine-year-old boy mimicking juvenile ossifying fibroma.

## 1. Introduction

Fibrous dysplasia is a developmental benign bone lesion characterized by the replacement of normal bone by excessive proliferation of cellular fibrous connective tissue which is slowly replaced by bone, osteoid, or cementum-like material [1]. The lesion presents itself in two forms: monostotic form, which denotes single bone involvement, mostly affects the cranium, often the occiput and the polyostotic form that denotes multiple bone involvement. Bones which bear the brunt are femur, tibia, ribs, and facial bones. It accounts for about 2.5% of all bone tumors and 7.5% of the benign bone neoplasms [2]. Hereby, we report a case of fibrous dysplasia which presented a rapid growth in a 9-year-old boy.

## 2. Case Report

**Figure 1:**
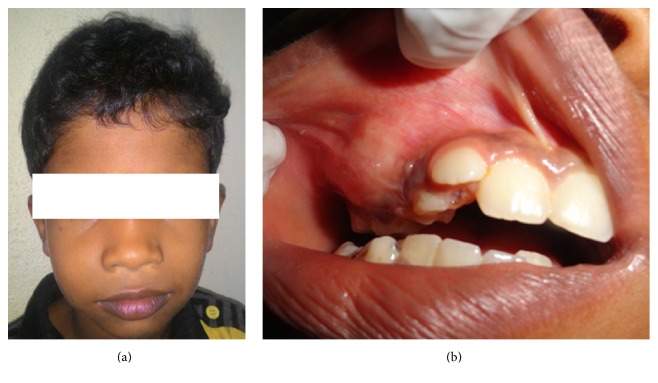
(a) Showing extraoral swelling in relation to the right side and (b) showing the extent of the intraoral ovoid swelling.

A medically fit nine-year-old male patient visited our dental department with a chief complaint of a swelling in relation to the right upper back tooth region since 3 months which was initially small in size but gradually grew up to the present size. There was no history of pain, pus discharge, or any other associated discomfort except for the unaesthetic facial asymmetry. His family and dental history were noncontributory. On examination, a diffuse, well-defined unilateral swelling measuring about 4 × 3.5 cm was seen on the right middle third of the face, extending superiorly from 1 cm below the infraorbital margin inferiorly to the inferior border of mandible, anteriorly from the ala of the nose, and posteriorly till the anterior border of the ramus. The skin over the swelling appeared normal (Figure 1(a)). On palpation, all the inspectoral findings were confirmed. The swelling was hard in consistency with well-defined borders. There was no tenderness, local rise in temperature, or paresthesia.

On intraoral examination, a solitary well-defined unilateral ovoid swelling was seen on the upper right buccal vestibule extending anteriorly from upper right front tooth region, posteriorly to upper right back tooth region measuring about 4 cm × 3 cm and not crossing the midline. The swelling appeared normal in colour with no surface changes. There was obliteration of the buccal vestibule, buccal cortical expansion, and slight palatal expansion (Figure 1(b)). On palpation, all the inspectoral findings were confirmed. The swelling appeared hard and nontender.

Based on the history and clinical presentation, a provisional diagnosis of juvenile ossifying fibroma in relation to the right buccal vestibule was given. Fibrous dysplasia was considered in differential diagnosis.

**Figure 2:**
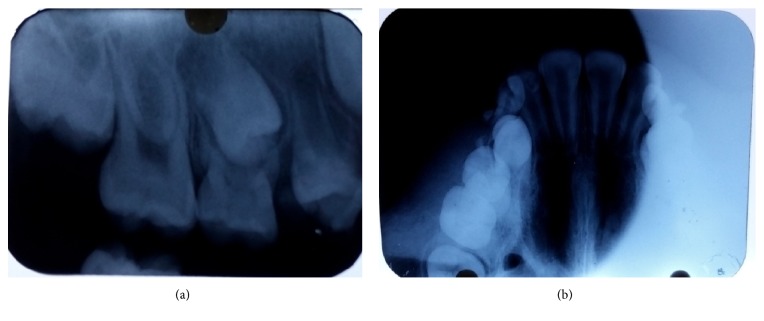
(a) Showing granular trabecular pattern in the IOPA of maxillary right back tooth region. (b) Showing maxillary true occlusal radiograph of same region with granular trabecular pattern, increased buccal cortical expansion, and mild palatal cortical expansion.

**Figure 3:**
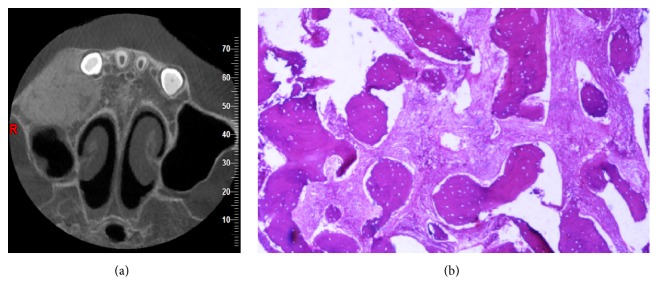
(a) Figure showing CBCT scan: axial section showing typical granular trabecular pattern and bicortical expansion with more than half of maxillary sinus involvement in relation to the maxillary right back tooth region. (b) Figure showing histological picture showing immature woven bone with fibrous stroma.

Intraoral periapical radiographic (IOPA) examination revealed radiolucency involving enamel, dentin, and pulp in relation to upper right posterior deciduous teeth and erupting premolars periapically. The trabecular pattern showed ground-glass appearance (Figure 2(a)). The maxillary true occlusal radiograph revealed increased radiopacity and buccal cortical expansion with ground-glass appearance. There was no sign of palatal involvement (Figure 2(b)). Cone beam computed tomography (CBCT) examination, axial section revealed expansion of the buccal and palatal cortical plates which had a well-defined margin with typical ground-glass trabecular pattern. More than half of the right maxillary sinus was involved (Figure 3(a)).

Based on the radiological findings, a radiological diagnosis of juvenile ossifying fibroma in relation to the maxillary right posterior region was given. Fibrous dysplasia was considered as radiological differential diagnosis, but the margin of the swelling was well-defined and well demarcated from the surrounding areas which was very much unlike FD whose margin is ill-defined and merges with the surrounding areas.

An incisional biopsy was performed which microscopically revealed irregularly shaped trabeculae of immature woven bone in a cellular, loosely arranged fibrous stroma. The bone trabeculae were not connected to each other and did not display any functional orientation (Figure 3(b)). All these features were suggestive of fibrous dysplasia. So taking into account the clinical, radiological, and histopathological examination, a final diagnosis of Monostotic Fibrous dysplasia in relation to the right maxillary posterior region was given.

## 3. Discussion

Fibrous dysplasia of bone was first described by Von Recklinghausen in 1891. In 1938, Lichtenstein and Jaffe first introduced the term fibrous dysplasia [3]. Fibroosseous lesions (FOL) represent a group of entities in which the normal bone is replaced by cellular fibrous tissue. Waldron in 1993 classified FOLs into three major groups, namely, fibrous dysplasia, cementoosseous dysplasia, and ossifying fibroma [4]. Later, a compendious classification was suggested by Eversole et al. in 2008 [5] who classified FOLs into bone dysplasia, cementoosseous dysplasia, inflammatory/reactive processes, metabolic disease, and neoplastic lesions. Several forms of fibrous dysplasia have been described. The monostotic form, characterized by the involvement of a single bone, which is the most common form. Polyostotic forms, characterized by the involvement of more than one bone, include three different types: (1) craniofacial fibrous dysplasia, in which the maxilla and adjacent bones are involved; (2) Jaffe's type (or Jaffe-Lichtenstein type), in which there is multiple bone involvement along with an irregular macular melanin pigmentation of the skin (café-au-lait spots); and rarely (McCune-Albright syndrome or Albright's syndrome, MAS), in which there is progressive bone involvement, at least one of the typical hyperfunctioning endocrinopathies and/or café-au-lait spots, with almost any combination possible. In MAS, fibrous dysplasia affecting the facial bone can be worsened when it is associated with acromegaly which is a rare manifestation of endocrine hyperfunction.

Fibrous dysplasia (FD) is classified as a benign fibroosseous lesion in which the normal bone is replaced with a fibrous connective tissue containing abnormal bone produced as a result of disturbance of bone metabolism [6]. The two dominant groups of benign fibroosseous lesions, ossifying fibromas and fibrous dysplasia, have a similar pattern of disease progression so it becomes irremissible to distinguish between the two [7]. A diagnostic clue for differentiating these two lesions is that ossifying fibroma has a well-circumscribed and sharply defined margin which is absent in case of FD.

FD is seen equally in males and females, with hugely varying phenotypes. In our case, a male patient was affected. FD affects the maxilla more than mandible and the frequent site of occurrence is the posterior region. Our case was also reported in the posterior maxilla. The polyostotic form is mostly seen in children younger than 10 years, whereas the monostotic form is found in a slightly older age group. In our case, the patient was 9 years old [8].

The etiology of FD suggests that it is caused as a result of postzygotic mutation of the alpha subunit of the guanine nucleotide (GNAS) binding protein alpha stimulating which in turn activates adenylate cyclase, thereby increasing the intracellular concentrations of cyclic adenosine 3′,5′-monophosphate (cAMP). This causes abnormal differentiation of osteoblasts and production of dysplastic bone [9].

FD is slow growing, but, in our case, the lesion presented with a rapid growth, which contradicted the typical clinical feature of fibrous dysplasia. The earliest clinical presentation of the disease is by a painless swelling of the jaws. Any bones can be affected by FD in a completely random distribution, even though buccal cortical bone was more affected in our case with mild palatal expansion [10].

FD lesions exhibit diverse trabecular patterns. Early lesions are more radiolucent than mature lesions and in few cases, the appearance of internal septa give rises to a multilocular appearance. The abnormal trabeculae are shorter, thinner, irregularly shaped, and more numerous, manifesting a granular appearance (“ground-glass” appearance, resembling the small fragments of a shattered windshield) as seen in our case. The other radiological manifestations are those resembling the surface of an orange (peau d'orange), a wispy arrangement (cotton wool), organization of the abnormal trabeculae into a swirling pattern similar to a fingerprint [11]. In our case, there was a well-defined radiographic margin which favoured a diagnosis of juvenile ossifying fibroma, unlike fibrous dysplasia which blends with surrounding bone [12].

Histopathological features include irregular trabeculae of woven bone, blending into the surrounding normal bone that lies within a cellular fibrous stroma. The varied shapes of the bony trabeculae resemble Chinese characters. All of these features was seen in our case too, suggesting a final diagnosis of Monostotic Fibrous dysplasia involving the right maxilla [1].

Facial FD that continues to expand in adults can be extremely deforming and has the highest rate of malignant transformation which can be very difficult define due to the changing nature of the lesions. The aim of the surgical therapy is to prevent pathological fractures and to reduce bone deformities. Only curative surgical recontouring was performed in our case and patient is being recalled to check for recurrence.

## 4. Conclusion

Differentiation of the FD lesions from juvenile ossifying fibroma is critical because the treatment protocols are entirely different in these two. Juvenile ossifying fibroma, although benign, is enucleated because it has a potential to recur, whereas fibrous dysplasia does not require treatment except when there is functional or aesthetic compromise.
