# Corrigendum to “Fibrous Dysplasia versus Juvenile Ossifying Fibroma: A Dilemma”

**DOI:** 10.1155/2018/5813805

**Published:** 2018-01-14

**Authors:** Sreelakshmi N. Nair, Raghavendra Kini, Prasanna Kumar Rao, Gowri P. Bhandarkar, Roopashri Rajesh Kashyap, Manjunath Rai, Neel Naik, Athul Santhosh

**Affiliations:** ^1^Department of Oral Medicine and Radiology, AJ Institute of Dental Sciences, Mangalore, Karnataka, India; ^2^Department of Oral and Maxillofacial Surgery, AJ Institute of Dental Sciences, Mangalore, Karnataka, India

In the article titled “Fibrous Dysplasia versus Juvenile Ossifying Fibroma: A Dilemma” [1], the name of the fifth author was given incorrectly as Roopashri Rajesh Kashyp. The author's name should have been written as Roopashri Rajesh Kashyap. The revised authors' list is shown above.
